# An Allergy to Goldfish? Highlighting Labeling Laws for Food Additives

**DOI:** 10.1097/WOX.0b013e3181c5be33

**Published:** 2009-12-15

**Authors:** Ian A Myles, Douglas Beakes

## To the Editor

Allergy to food additives is a well-documented phenomenon in the literature. Annatto is an orange food coloring that can be found in both a synthetic or a natural form [1]. If natural, it is derived from the fruit of the *Bixa orellana *tropical bush [2]. It is considered a carotenoid because of its chemical similarity to beta-carotene [3]. This natural form is often used in the regional cuisine of Caribbean and Latin American cooking. In processed foods annatto is most often used as a coloring for butter, cheeses, and other snack foods [4]. Whereas European laws mandate that food colorings be clearly labeled on all packaging [5], laws in the United States allow for an "artificial color" disclaimer if the additive is in the synthetic form or a "color added" disclaimer when in its natural form[2].

The following case illustrates how the exemptions provided for food additives with allergenic potential endanger consumer safety and provide difficult challenges for diagnosis.

A 2-year-old female with no past medical history presented to the allergy clinic for evaluation of recurrent hives associated with various food ingestions. At the age of 8 months, the patient began having perioral and anterior neck redness 10 to 15 minutes after eating. The reactions would occur only after meals but there was no obvious food category that the child would react to on a consistent basis. The reactions were not associated with the child's activity level after food intake was complete. These reactions progressed, during a 6-month period, to immediate symptoms of hives on the abdomen and thighs along with angioedema of the lips. There were no respiratory, gastrointestinal, or upper airway symptoms. The mother performed her own elimination diet over the course of several months and ultimately limited the patient's diet to fruits, vegetables, rice, cow's milk, and chicken. On the limited diet, the patient's symptoms completely resolved. The mother attempted reintroduction of cheese, yogurt, crackers, and pastas, beginning with foods targeted to children with food allergies (labeled as soy-free, wheat-free, dairy-free, etc). The patient tolerated these introductions intermittently; she was able to eat one product containing cheese, for example, without issues but not a different cheese product. However, if she reacted to a specific brand and flavor once, then she would consistently react to future exposures. The patient continued to have full-body hives and angioedema without anaphylaxis to foods and the mother continued to limit specific products but was frustrated with the lack of an obvious trigger in the foods that were being avoided.

At the time of clinical evaluation, the physical examination, including vital signs, was normal. The child had no evidence of eczema, no wheezing on examination, no dermatographism, no urticaria, and no abnormalities of the nasal mucosa. The patient's primary pediatrician had drawn a complete metabolic panel and complete blood count with differential, both of which were entirely within normal limits.

**FIGURE 1:**
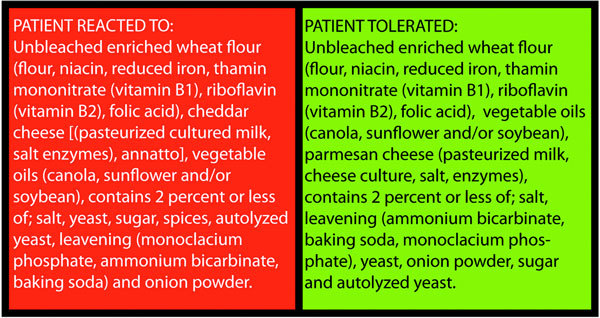
**A reproduction of the ingredient panels from the cheddar flavored cracker that the patient reacted to (red panel) and the parmesean flavor of the same brand cracker that the patient tolerated (green panel)**.

At 20 months of age, during the mother's continued diet modification campaign, a breakthrough occurred when the mother noted that the patient tolerated a parmesan-flavored snack cracker but did not tolerate the cheddar flavor of the same cracker from the same company. Before presenting to our clinic, and unaware of the dangers, the mother fed the patient various products containing the ingredient she suspected on at least 4 occasions with the same immediate onset of symptoms. An immediate reaction could be reproduced with as little as 2 of the small snack crackers or 1 bite from various other previously implicated dishes. At this point the mother collected all of the products that the patient had and had not reacted to in her home experimentation and presented to our clinic for further evaluation. By comparing food labels, we confirmed the mother's suspicion that annatto was the only ingredient shared by the food the patient would react to that was lacking in the foods that she tolerated. The reproductions of the labels from the parmesan and cheddar crackers are in Figure 1.

The patient had a normal baseline tryptase level and a normal total IgE. In vitro laboratory evaluation with Immunocap to annatto dye is no longer commercially available. Skin testing in this case is of unknown predictive value. In a previous case report [6], an adult patient was described with anaphylaxis to annatto food dye. In the workup both skin test and in vitro sensitivity were identified. However, in the 3 other documented patients with immediate onset reactions, skin testing was negative [4,7].

Because the patient is still younger than 3 years and the mother has full control over the patient's diet, she declined further evaluation at this time. The plan is for the patient to return to our clinic before her entering school to undergo skin testing with both the "prick-prick" method from the snack crackers softened in water and commercial extract that can be obtained from various cereal and snack companies followed by an open challenge. This will be done only if she is more than 12 months out from any suspected reaction to annatto and with the understanding that the predictive value of the skin test is not known. The family was given self-injectable epinephrine along with instructions to request a repeat serum tryptase from her emergency physician if another attack should occur. Therefore, we consider the diagnosis to have been made clinically with the acknowledgment that skin testing or in vitro positivity would further solidify the diagnosis.

Although there are rare case reports of allergies and anaphylaxis to annatto dye intake, this case illustrates the complexity that allergies to food additives present [6,7]. Although only the natural formulation of annatto has been documented as allergenic, there are documented allergic reactions to other synthetic carotenoids and, therefore, synthetic annatto has a theoretical potential to induce allergic responses [2,8]. Personal communication with the manufacturers of the products our patient reacted to revealed that the annatto each manufacturer uses is a natural formulation. However, because of exemption from FDA labeling law Title 21CFR-70.25, annatto dye can be lumped into an "artificial color" disclaimer on the package label if it is in the synthetic form or a "color added" disclaimer when in its natural form [2]. Other color additives that meet this exemption, and are therefore also considered optional for separate ingredient listing, include beta-carotene, beet powder, caramel color, carrot oil, carmine, fruit juices, paprika, riboflavin, saffron, turmeric, and vegetable juices [7]. Nonexempt colorings must be listed individually on the label.

In Europe, annatto is given the designation E160b if not spelled out. The European Union Directive 2000/13/EC established a list of food ingredients that require clear labeling [3]. Annex II of the Directive established a list of additives that must be listed by category and the additive name, including food colorings, flavor enhancers, preservatives, and others [8]. This was consistent with the Codex of General Standard for the Labeling of Prepackaged Foods article 6 and 8.1.13 for member countries of the United Nations and World Health Organization [9]. At the request of the European Food Safety Authority, Directives 2005/26/EC and 2005/63/EC allotted time for companies to test the allergenicity of certain ingredients including annatto and related carotenoids [3]. The dead-line for proof of safety passed in November 2007, and no further exemptions have been offered to food colorings like annatto in the most recent European Union proposals that have yet to become directives [10].

However, there are still dangerous limitations in the European labeling laws for individually wrapped foodstuffs. European laws do not require either ingredient listing or allergen warnings on foodstuffs in packages that have less than 10 cm[2] or surface area [9]. This lack of identification on individually wrapped items has been implicated in at least 1 fatality of a patient allergic to nuts [9]. It should also be noted that whereas European laws may make it easier for an allergist to uncover a food additive allergy, consumers in Europe continue to find frustration in interpreting food labels. In one study [11], parents of children with food allergies and adults with similar food allergies reported labels to be difficult to read because of font size, contrast, and/or jargon. They also reported inconsistencies in the labels because of changes in recipes, incorrect language translation, and even ambiguity in symbols meant to convey allergenic content. Although some regional differences were present, the final conclusions strongly highlighted the need for improvement on labeling laws to better consumer understanding and safety.

Reactions to another food coloring, carmine, along with many other additives have been reported in an array of both IgE and non-IgE-mediated reactions [2]. Of note, a mounting number of reported carmine reactions prompted a US Food and Drug Administration amendment to the Code of Federal Regulations requiring that carmine always be listed separately on food or cosmetic labels. However, the amendments will not take effect until January 2011 [1]. No such changes have been proposed for annatto in the United States.

Reactions to flavorings can present a difficult challenge for allergists because US labeling laws do not require the listing of flavoring additives as a means to protect the proprietary recipes of manufactures [2]. The potential for severe reactions to food additives makes a strong case for more ready access to diagnostic testing to these compounds and reconsideration of the US labeling laws to mandate that all known allergenic additives be listed separately. Also, work is needed to close the knowledge gap as to the minimum dose of these additives required to elicit allergic response because such information would be greatly beneficial to all interested parties [12].

Although this patient clearly benefited from the diligent investigation conducted by her mother, it was only due to voluntary listing of annatto by the food manufacturers that allowed for a diagnosis in this case. Interestingly, apart from translation, the packages for the foodstuffs in question are not different between the US and European nations. The corporate offices of these companies each stated, in personal communications, that their decision on the ingredients to list separately is based on the region with the strictest regulations, and then for ease of packaging all of the products are labeled in a similar manner. Therefore, products sold only in the United States or by companies that elect to relax their labeling because of the less stringent regulations provide a unique challenge for patients with allergies to food additives. The important points raised by this letter include the necessity of understanding the labeling laws in one's region and the high level of suspicion required to diagnose allergies to food additives, as it is foreseeable that patients could have a negative workup to the primary allergens in the foods to which they reacted and then be provided with false assurance that they are nonallergic.
